# Modified Oblique Lobenhoffer (MOL) approach for posterolateral and posteromedial column access in tibial plateau fractures: a detailed cadaveric anatomical study

**DOI:** 10.1186/s10195-024-00769-z

**Published:** 2024-05-20

**Authors:** Juan Boluda-Mengod, Beatriz Olías-López, Pau Forcada-Calvet, Azucena Martín-Herrero, Mario Herrera-Pérez, Javier Álvarez-De-La-Cruz, Alejandro Herrera-Rodríguez, José Luis Pais-Brito

**Affiliations:** 1 Orthopaedic Trauma Unit, Department of Orthopaedics, Hospital Universitario Canarias (HUC), Carretera Ofra S/N, 38320 La Laguna, Tenerife Spain; 2 Department of Orthopaedics, Hospital Universitario Canarias (HUC), Carretera Ofra S/N, 38320 La Laguna, Tenerife Spain; 3 Faculty of Medicine, Universidad de La Laguna (ULL), Campus de Ofra, S/N, 38071 La Laguna, Tenerife Spain; 4 Department of Anatomy, Faculty of Medicine, Universitat de Lleida (UdL), Avinguda de L’Alcalde Rovira Roure, 80, 25198 Lleida, Spain

**Keywords:** Tibial plateau fracture, Posterolateral column, Surgical technique, Surgical approach, Lobenhoffer approach, Modified Oblique Lobenhoffer, Main Deformity Direction

## Abstract

**Background:**

Tibial plateau fractures involving posteromedial (PM) and posterolateral (PL) columns are complex injuries that require an appropriate approach. The management of the PL column in these cases can be controversial, and limitations using deep posteromedial interval approaches have been referenced. In this paper, a modification of the Lobenhoffer approach, designed to optimize the access to the PL column, is described in detail. The aim of this study was to assess the feasibility of this approach in a cadaveric anatomical study.

**Materials and methods:**

In total, five fresh-frozen cadaveric specimens were used for detailed anatomical study surrounding the approach. Relationships with cutaneous and deep neurovascular structures were evaluated. The exposure area of the PL and PM columns using this approach was assessed.

**Results:**

The cadaveric study showed safe and adequate exposure. Oblique skin and fascia incision just medial to the posterior midline was safe to protect the medial sural cutaneous nerve and the small saphenous vein. Elevation of the popliteus and tibialis posterior muscles offered safe protection of the anterior tibial artery and popliteal neurovascular bundle during retractor placement. Adequate full proximal exposure of the PM and PL columns, including the posterolateral lateral (PLL) and posterolateral central (PLC) segments, was obtained in all specimens.

**Conclusions:**

The Modified Oblique Lobenhoffer (MOL) approach can be a feasible option to access PL and PM columns in tibial plateau fractures.

**Level of evidence:**

IV.

## Introduction

Tibial plateau fractures that involve the posteromedial (PM) and posterolateral (PL) columns are complex injuries that require an appropriate approach for reduction and fixation [1–6]. The most common way to address the PM column is through different types of deep posteromedial interval approaches between the medial gastrocnemius and the pes anserinus. These have been described in literature with different patient positions and skin incisions [2, 3]. The most commonly used are the posteromedial approach in supine position [7], Lobenhoffer approach (straight incision) [8, 9] and Luo approach (posterior reverse L-shaped approach) [1, 10] in prone position. However, the ideal surgical strategy to address the PL column in fractures involving the PM and PL columns can be controversial [11–20]. Several authors refer to limitations with the PL column exposure, reduction and fixation using the Lobenhoffer or Luo approaches, and recommend to associate a PL approach [11, 12]. In this sense, various approaches, including extended anterolateral, lateral or posterolateral incisions, have been described to specifically address the PL column [3, 4, 20–28]. However, these approaches lack access to the PM column and also come with their own limitations and risks [13, 22, 29]. Other authors have proposed direct posterior approaches between gastrocnemius muscles to address both PL and PM columns, but requiring more complex dissection [30–33].

This paper describes, in detail, a modification of the Lobenhoffer approach, designed to optimize access to the PL column through a deep posteromedial interval approach [4]. The aim of the study was to assess the feasibility of the Modified Oblique Lobenhoffer (MOL) approach in a cadaveric anatomical study.

## Material and methods

### Cadaveric study

**Fig. 1:**
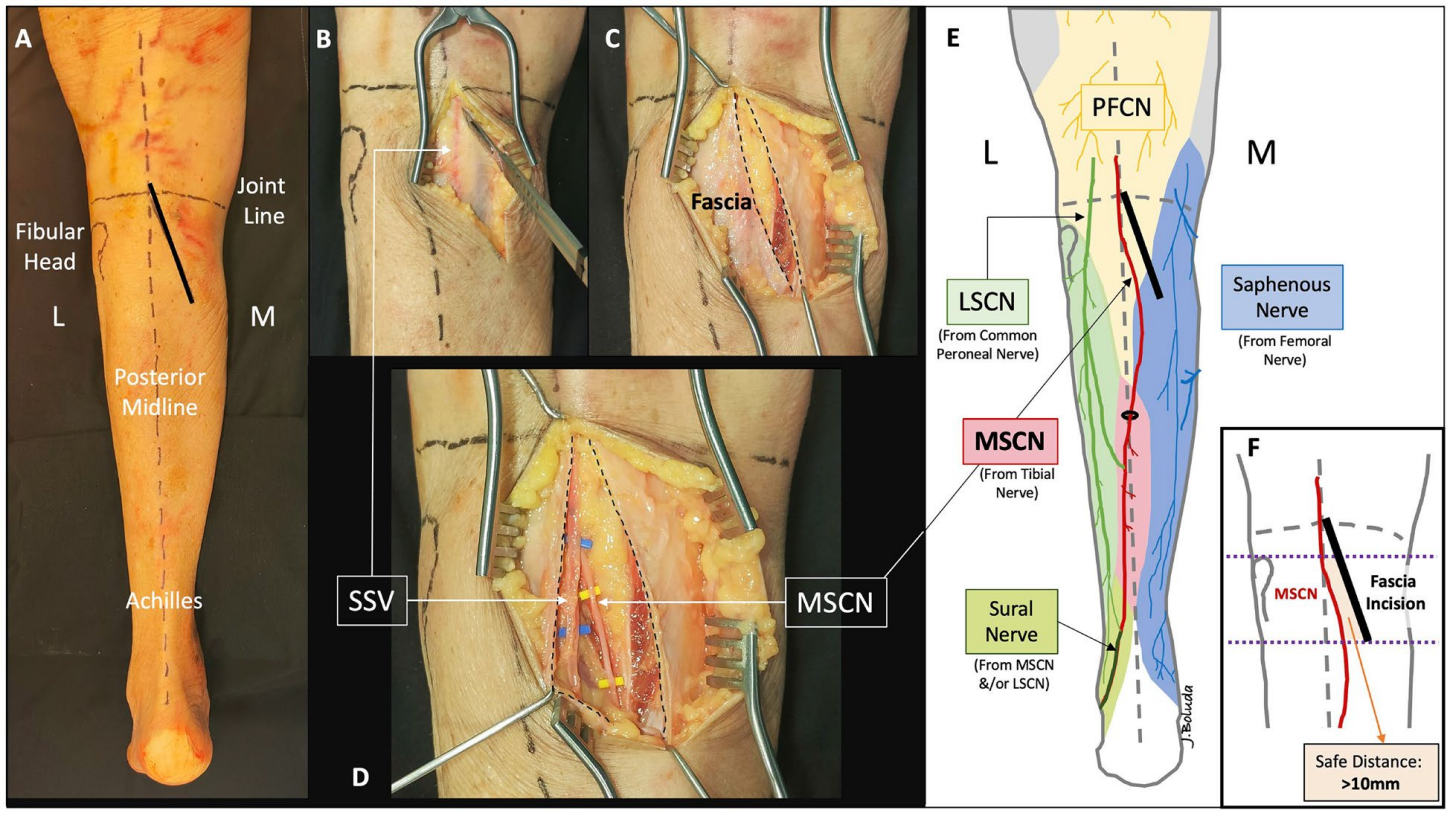
Step 1: skin and fascia incision. **A** Landmarks in a cadaveric left leg. Oblique skin incision near to posterior midline. L: lateral, M: medial. **B** Medial gastrocnemius fascia incision near to posterior midline, starting medial to the small saphenous vein (SSV; recognized by translucency). **C** Oblique fascia incision (dotted lines). **D** Anatomic detail retracting the fascia towards lateral (not necessary for the clinical surgical approach) to expose the anatomic location of the SSV (blue) and the medial sural cutaneous nerve (MSCN; yellow) under the fascia. Note the position of the MSCN under the SSV and located at the posterior midline at the level of the joint line and first centimetres below. The obliquity of the skin and fascia incision is parallel to the path of the MSCN. **E** Schematic representation of cutaneous innervation areas (coloured) and cutaneous nerves (lines) in relation with the approach. Black circle represents the passage of the MSCN through the fascia to the subcutaneous. PFCN: posterior femoral cutaneous nerve, LSCN: lateral sural cutaneous nerve. Note the MSCN distal trajectory to lateral forming the sural nerve (careful retraction to lateral is safe during the approach). **F** Diagram representing the distance between the fascia incision and the location of the MSCN, measured at and below the level of fibular head. Definition of ‘safe distance’ was > 10 mm

**Fig. 2:**
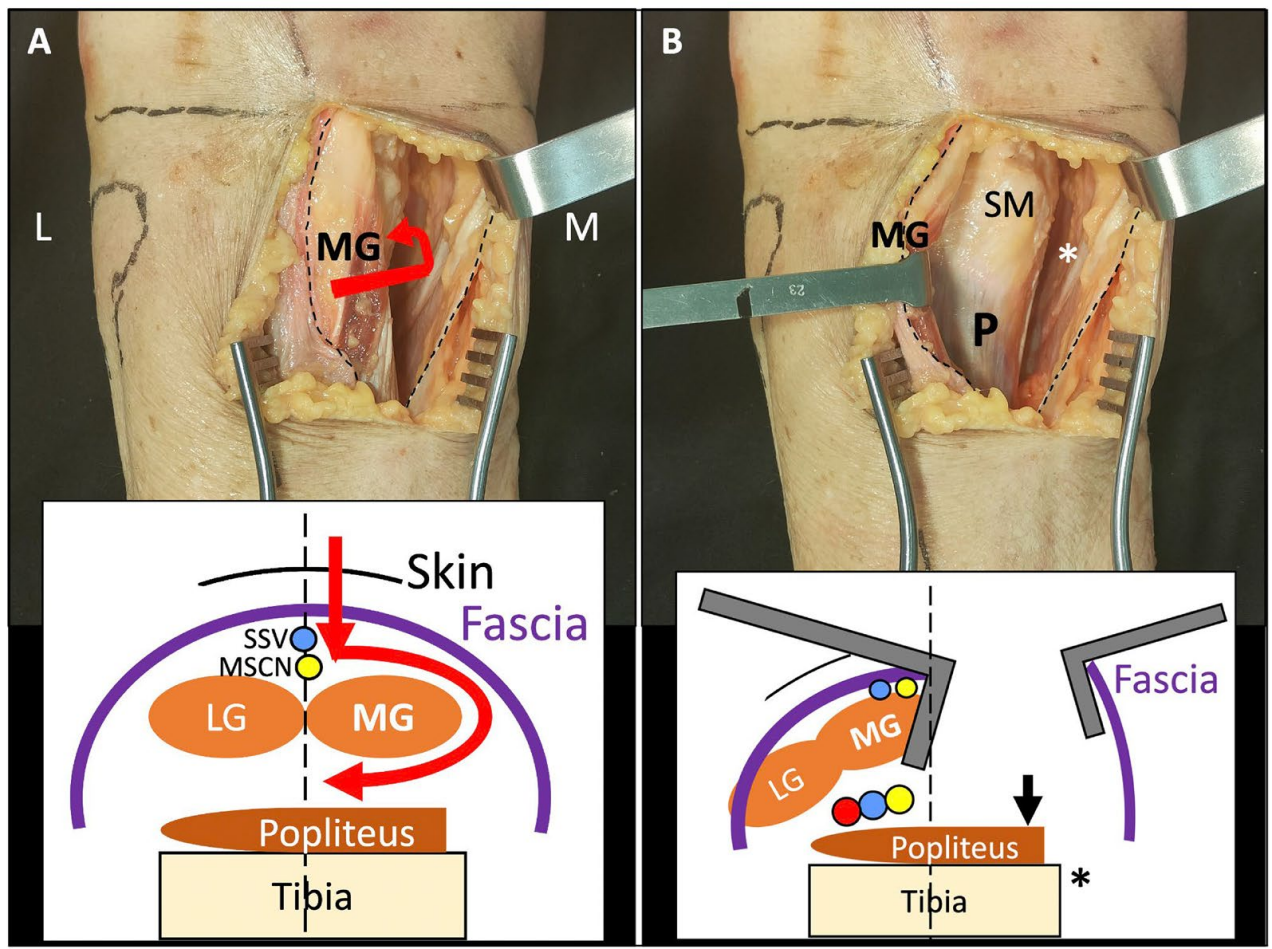
Step 2: deep posteromedial interval. **A** Medial blunt dissection in the space between the fascia (dotted lines) and the medial gastrocnemius (MG) muscle. Schematic axial view representation of skin and fascia incision near to posterior midline and the dissection between fascia and muscle (red arrows). SSV: small saphenous vein, MSCN: medial sural cutaneous nerve, LG: lateral gastrocnemius. **B** Lateral retraction of the MG muscle exposing the fascia over the popliteus (P) and semimembranosus (SM) muscles. Asterisks: pes anserinus. Schematic representation of the MG muscle retraction

**Fig. 3:**
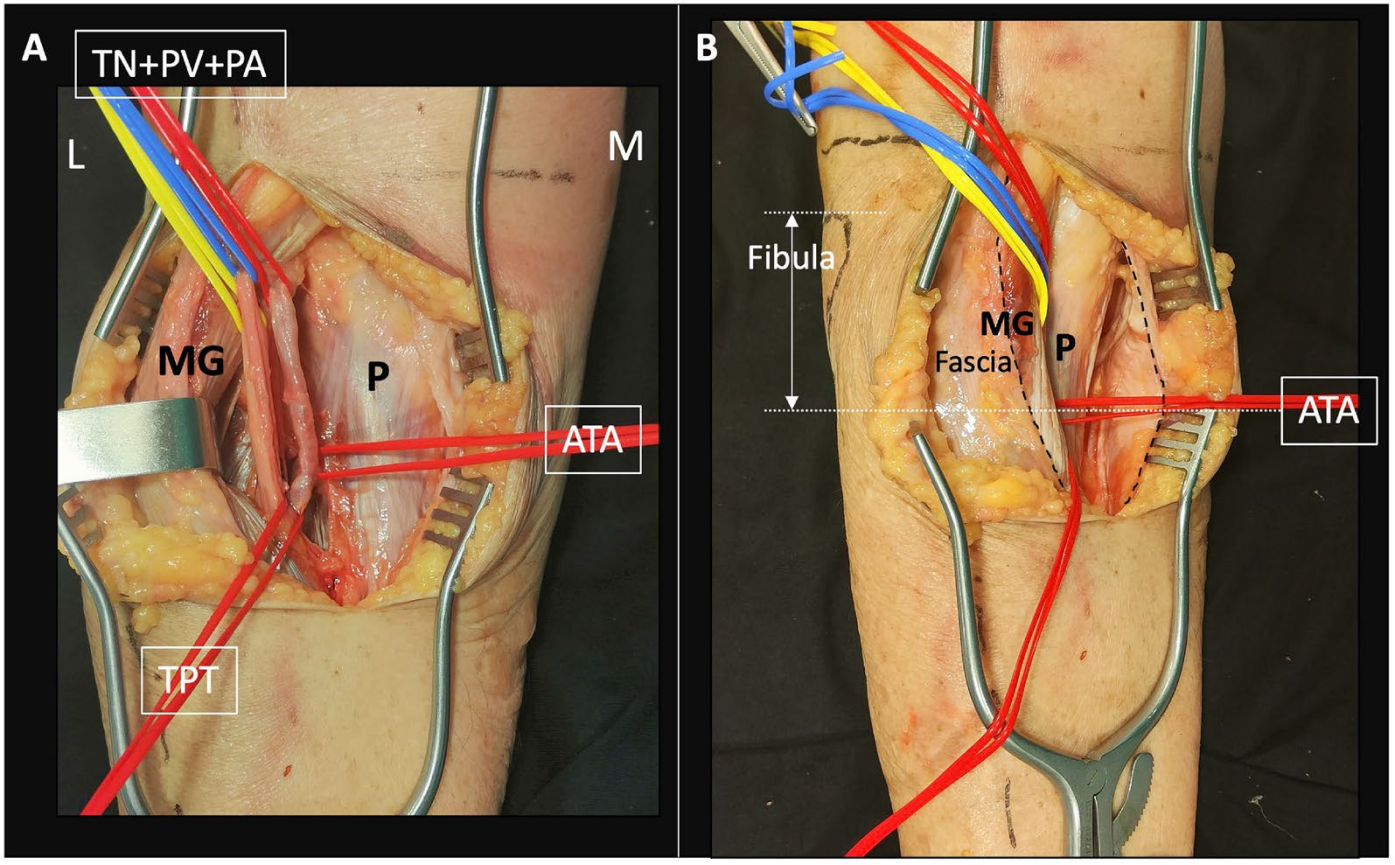
Anatomical details (not necessary for surgical approach). **A** Oblique view. Popliteal neurovascular bundle in the interval between the medial gastrocnemius (MG) and the popliteus (P). TN: tibial nerve, PV: popliteal vein, PA: popliteal artery, ATA: anterior tibial artery, TPT: tibio-peroneal trunk. **B** References of the location of the neurovascular bundle in the surgical approach. Note the level of the ATA in relation to the fibular head, relevant for the first lever retractor placement

**Fig. 4:**
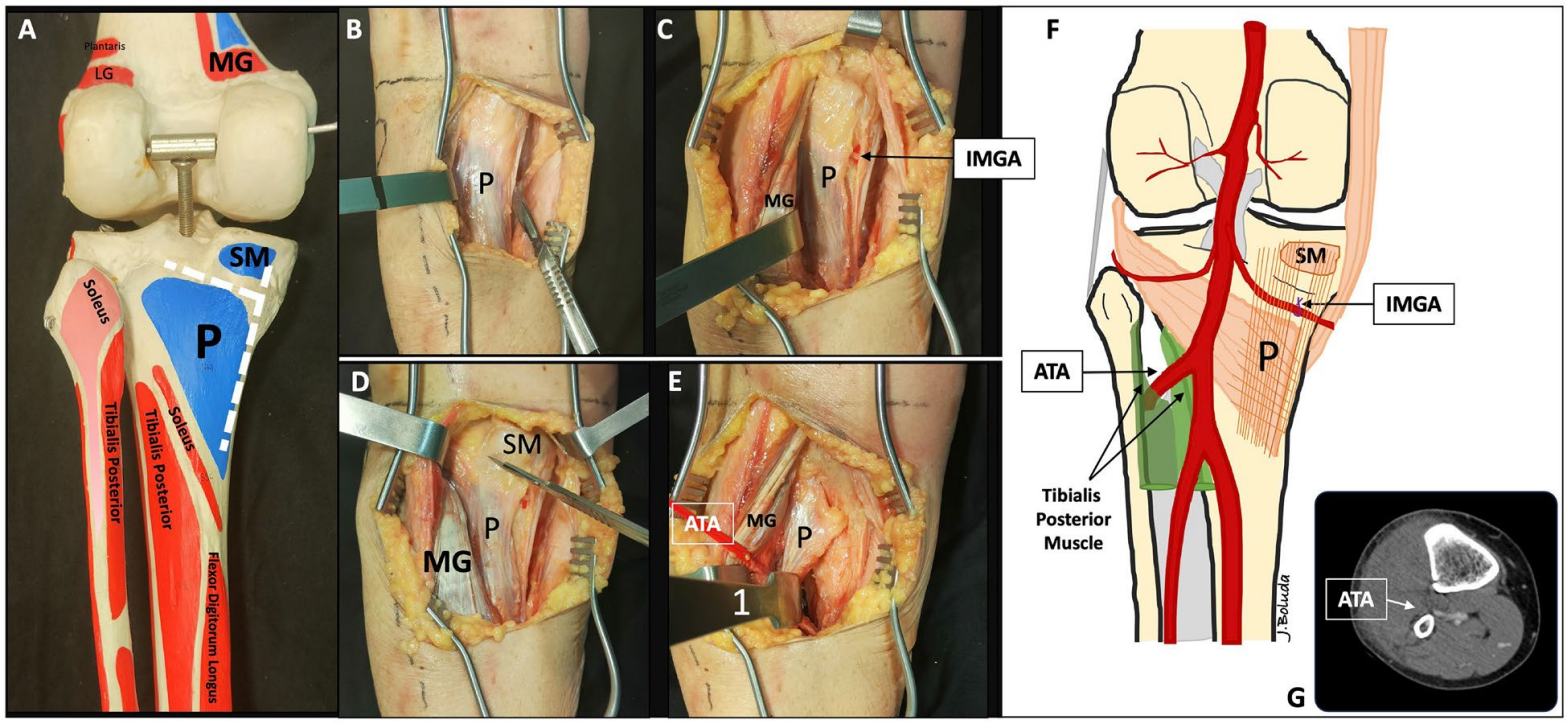
Step 3: popliteus elevation. **A** Reference of the T-shape fascia incision in an anatomic model. The insertion of the popliteus (P) and the semimembranosus (SM) muscles are represented in blue. **B** Longitudinal incision of the popliteus–semimembranosus fascia over the medial edge of the popliteus. **C** Exposure of the inferior medial genicular artery (IMGA) under the fascia and located at the proximal border of the popliteus muscle. Identification and ligation of this vessel is recommended. **D** Transverse oblique fascia incision between the proximal border of the popliteus muscle and the semimembranosus. Careful incision is recommended to avoid damaging the IMGA under this fascia. **E** Subperiosteal lateral reflection of the popliteus muscle and the tibial origin of the tibialis posterior muscle. First blunt Hohmann retractor (1) over the lateral edge of the tibia. The red vessel-looped references the anterior tibial artery (ATA) between the two heads of tibialis posterior muscle, for anatomical reference (not necessary for surgical approach). **F** Schematic representation of the arteries and its relationships. IMGA is under the popliteus fascia while popliteal artery, and normally the ATA, are over the fascia. Note that when IMGA is ligated, careful lateral retraction of the vascular structures is safe during the approach. Note that the ATA is partially protected by the tibial origin of tibialis posterior muscle after subperiosteally dissection is performed, when the retractor is placed in the lateral edge of the tibia. **G** Angio-computed tomography (CT) example illustrating the anatomical position of the ATA crossing in axial view, demonstrating its proximity to the fibula rather than the tibia

**Fig. 5:**
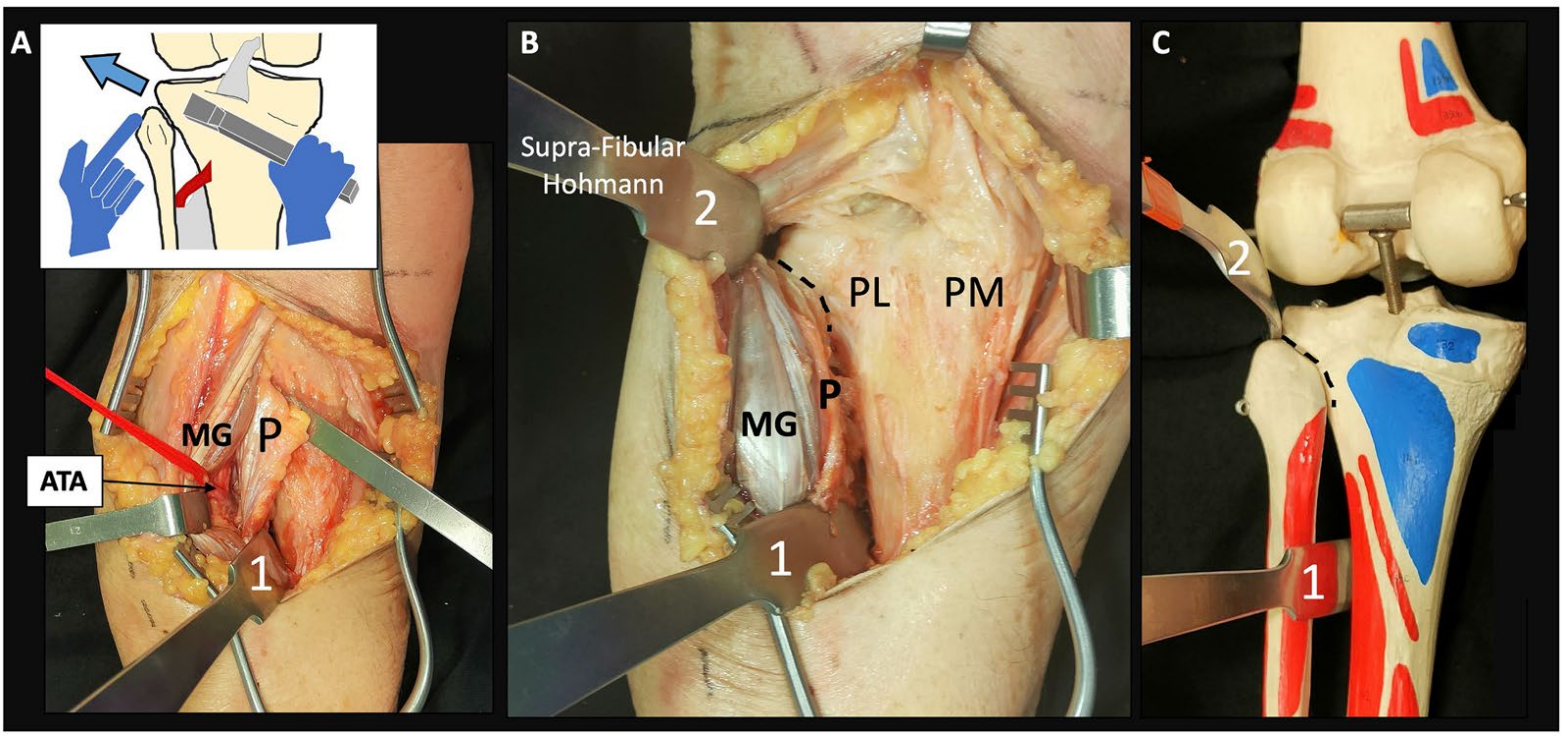
Step 4: posterolateral column exposure. **A** Subperiosteal dissection under the popliteus (P) in a direction towards the lateral aspect of the tibial plateau over the fibular head. Palpation of the skin over the fibular head with the other hand facilitates spatial orientation and appropriate periostotome direction. Distal blunt Hohmann retractor (1). ATA: anterior tibial artery. **B** Exposure of the posterolateral (PL) and posteromedial (PM) columns after the proximal supra-fibular Hohmann retractor (2) is in place. Normally a bent pointed Hohmann is preferred (see Figs. 6 and 10). Complete detachment of the periosteum is performed to demonstrate the potential bony surface exposure (not recommended for clinical surgical approach). Dotted lines represent the tibiofibular joint. **C** Location of the Hohmann retractors in an anatomical model

In total, five fresh-frozen cadaveric specimens were selected for detailed anatomical study (entire leg from groin to foot). They were all adult specimens, three were female and two were male, with four left legs and one right leg. The average age at death was 84 (range 78–91) years. Exclusion criteria were previous knee injuries or surgeries, poor preservation conditions and skin abnormalities (such as tattoos or scars). Cadaveric dissections and observations were performed by two orthopaedic surgeons with experience in anatomic dissections. The MOL approach was performed in prone position. Relevant neurovascular details surrounding the MOL approach were evaluated and photographed. The location of the medial sural cutaneous nerve (MSCN) was assessed with respect to the fascia incision and the posterior midline. A measurement of 10 mm or more between the fascia incision and the MSCN, at the level of the fibular head and below, was defined as a correct ‘safe distance’ (Fig. 1). The popliteal neurovascular bundle, the anterior tibial artery (ATA) and the tibioperoneal trunk were identified and dissected. The location and relationships between the ATA and surrounding structures were evaluated to determine the safety of the distal retractor placement. The exposure area of the PL and PM columns using this approach was evaluated. The criterion for ‘complete exposure area’ was defined when the entire proximal aspect of the posterior cortex of the tibial plateau was accessible from corner to corner, including the proximal posterolateral part of the tibia over the fibular head. Images from a cadaveric specimen (left leg) are presented in this paper to show the MOL approach, related anatomical details and exposure (Figs. 1, 2, 3, 4 and 5).

### Surgical technique and anatomic details

**Fig. 6:**
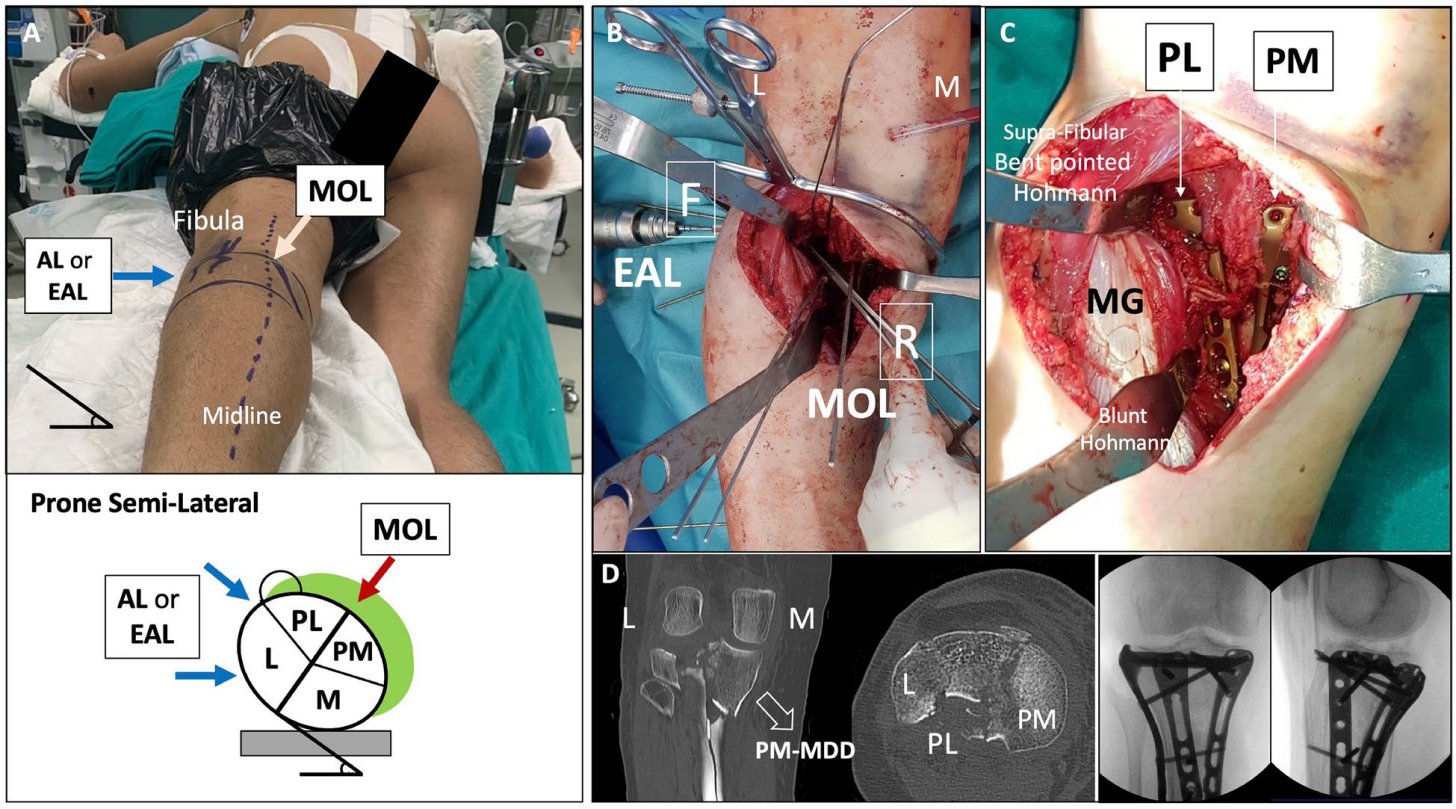
Clinical case example of the MOL approach and simultaneous global fracture control in a multi-columnar PM-MDD fracture. **A** Prone semi-lateral fixed position (left leg) allowing simultaneous access through the MOL and anterolateral (AL) or extended anterolateral (EAL) approaches. Diagram representing the position (tilted around 40°) in axial view, and the level of the skin incision (red arrow) and exposure (green area) of the MOL approach. **B** Reduction (R) of the PL column fragment with a 90° dissector forceps, under direct visualization through the MOL approach. Simultaneously, provisional and definitive fixation (F) of the articular PL fragment was performed through the EAL approach (pre-fibular window). Note also simultaneous reduction and provisional fixation of posteromedial and lateral columns in this position. **C** Final reduction of the posterolateral cortex and fixation with a posterolateral (PL) plate. Posteromedial (PM) plate in the proper location to counteract the PM-MDD. Note the location of the proximal bent pointed Hohmann retractor at the lateral aspect of the tibial plateau, over the fibular head. In this image, the distal blunt Hohmann rests on the PL plate to relax the tension. **D** Pre-operative computed tomography (CT) scan and intraoperative fluoroscopy

**Fig. 7:**
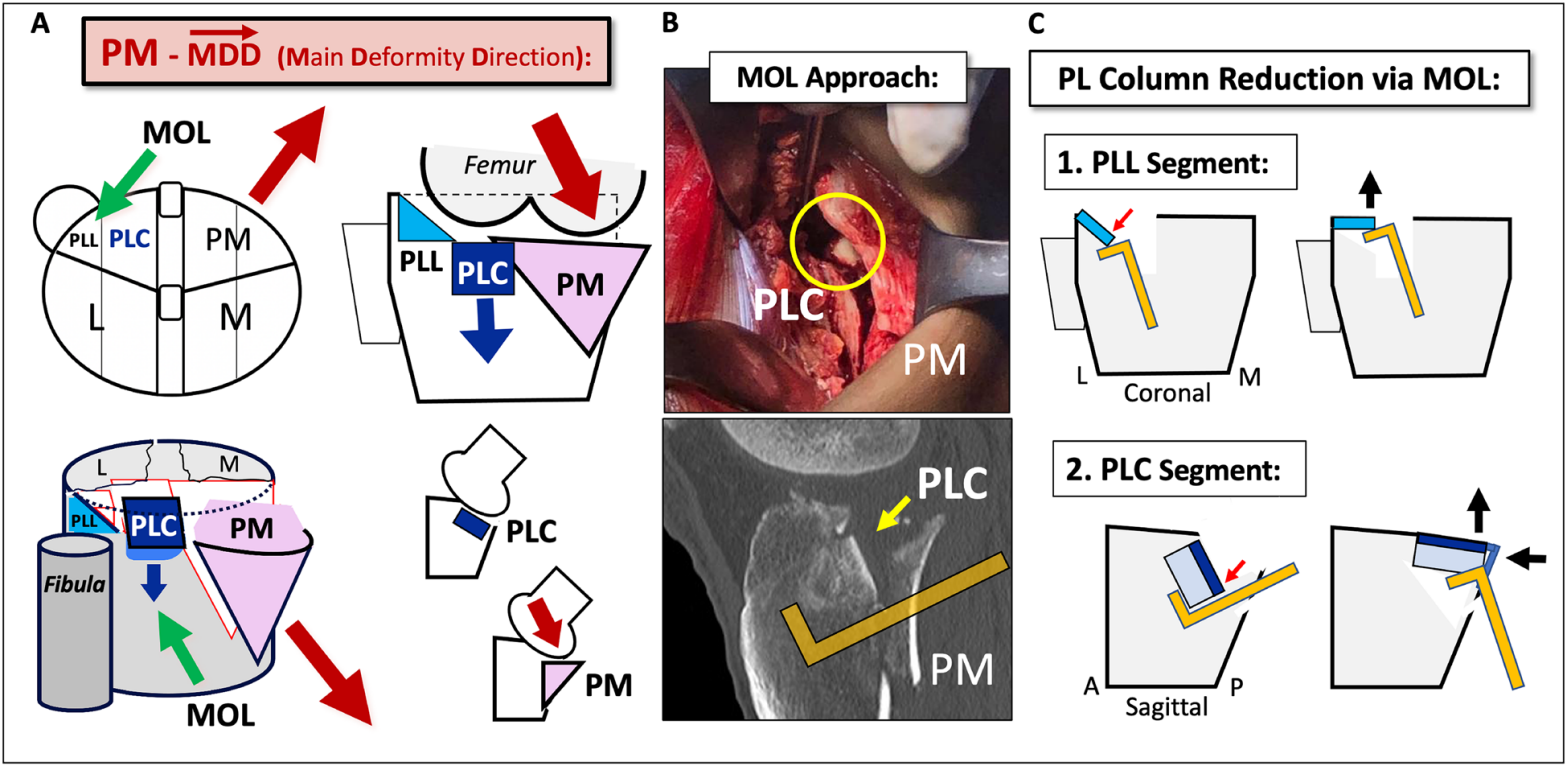
Typical deformity of the PL and PM columns in a multi-columnar PM-MDD fracture and reduction of the PL column through the MOL approach. **A** Schematic representation of global displacement of the columns in a PM-MDD (red arrows). The posterolateral central (PLC) segment is more affected and depressed than the posterolateral lateral (PLL) segment. Posterior angular displacement of the PLC fragment in the sagittal plane and medial angular displacement of the PLL fragment in the coronal plane. Green arrows represent the direction of management through the MOL approach, counteracting the MDD and managing the PL fragment deformities. **B** Case example of the ‘posterior split-depression’ concept (PM column split fracture and PL column depression). Surgical view with the MOL approach: visible articular fragment of the PLC (yellow circle) through the PM fracture window. Sagittal CT scan and strategy of reduction of the PL fragment (yellow arrow) before fixing the PM column. **C** Schematic representation of PL column reduction via MOL in typical deformities of the PLL and PLC segments with a 90° dissector forceps to elevate fragments in the proper direction

**Fig. 8:**
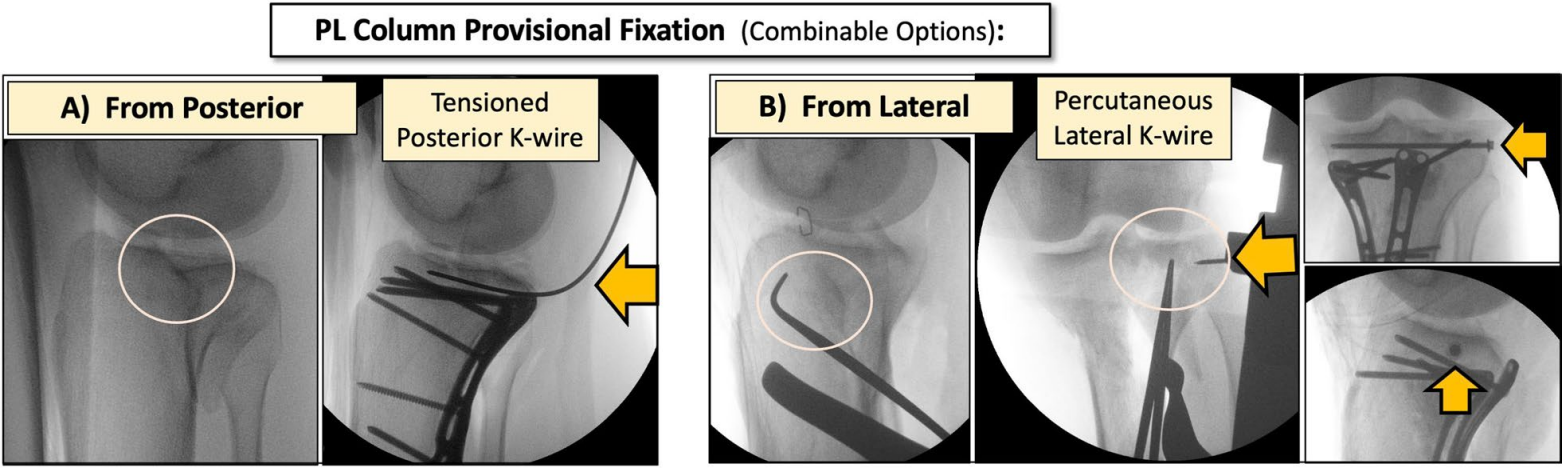
Options for PL column provisional fixation after reduction from the MOL approach. **A** From posterior: case example of provisional fixation using a tensioned K-wire under the elevated PL fragment. Subchondral K-wire position allows PL plating below the K-wire. **B** From lateral: case example of a PL fragment reduction from MOL (with a 90° dissector forceps) with simultaneous provisional fixation using a percutaneous lateral K-wire and a percutaneous lateral screw

**Fig. 9:**
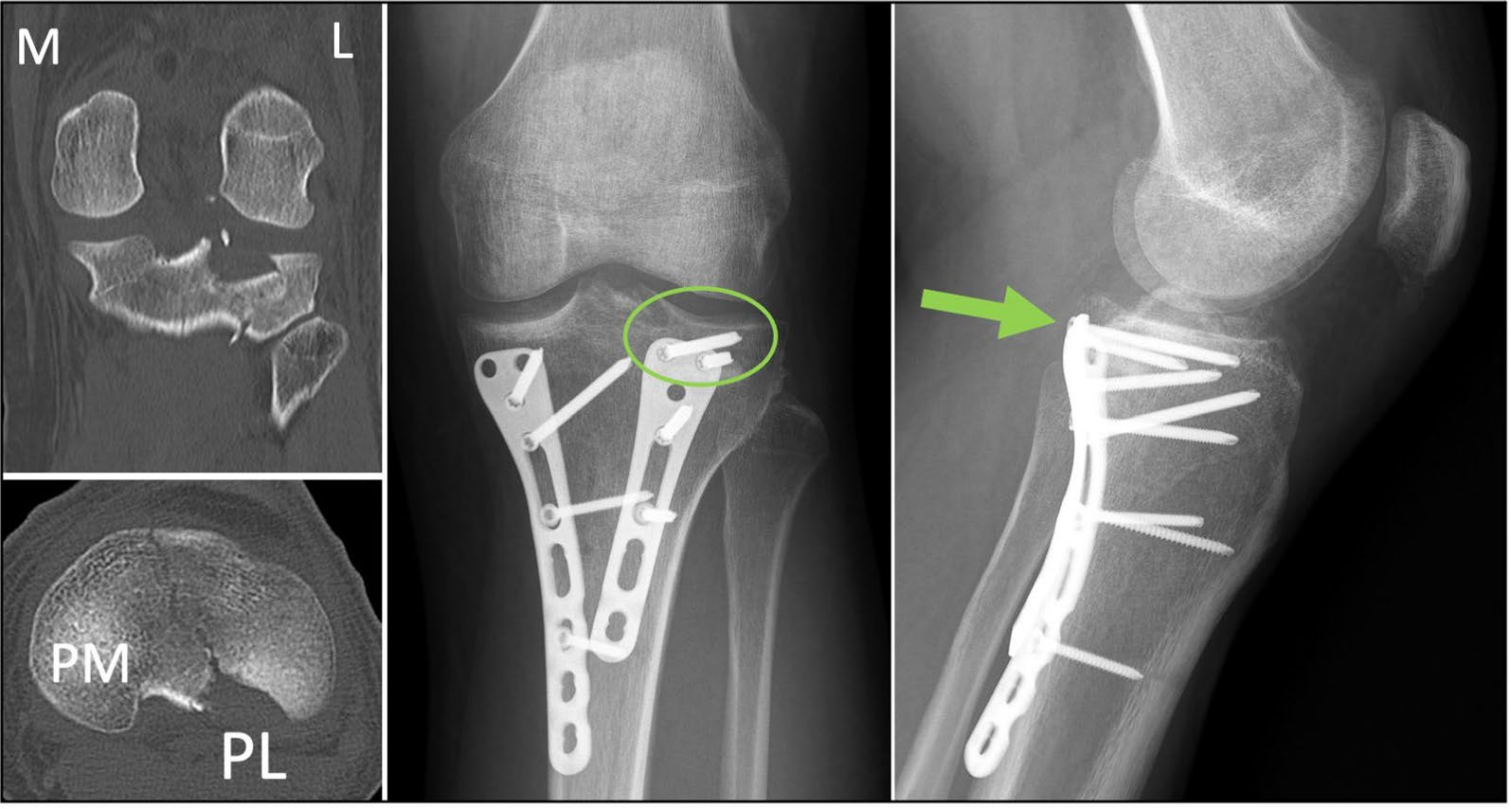
Case example of adequate screw trajectory in the proximal holes of the posterolateral plate using a MOL approach in a two-column PM + PL fracture

**Fig. 10:**
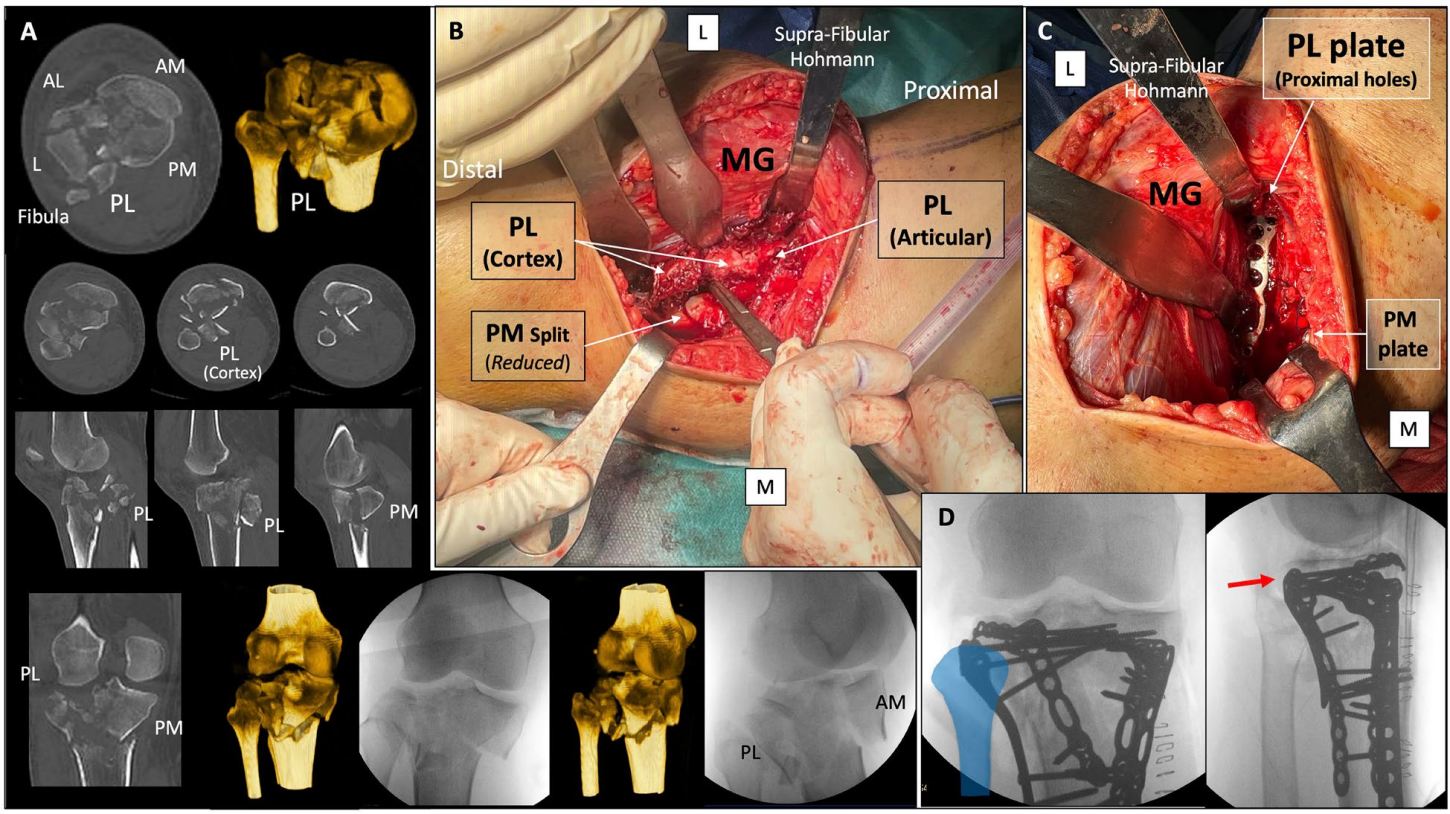
Clinical case example of PL column exposure and PL plate fixation via MOL approach in a complex multi-columnar fracture in a 43-year-old man with bulky gastrocnemius. **A** Pre-operative CT scan and fluoroscopy. Note the comminution and the displacement of the PL cortex fragments. **B** Exposure of the PL and PM columns using the MOL approach. Note the location of the supra-fibular Hohmann and that the full exposure of the PL column was achieved from the PL articular level (including the PL corner) to the most distal cortical fragments. PM column split is partially reduced in this image. **C** Surgical image of the PL plate location and the feasibility for proximal screw placement near to the supra-fibular Hohmann. The distal Hohmann rests on the PL plate to avoid unnecessary tension. PM plate is partially visible in this image. **D** Intraoperative fluoroscopy. Fibula marked in blue. Proximal screw of the PL plate (red arrow) was feasible with an appropriate direction through the MOL approach. In this case, simultaneous fracture control was performed in a fixed prone semi-lateral position, combining an anteromedial approach (flexing the knee for anteromedial reduction and plating), the pre-fibular window of the extended anterolateral approach (lateral plate and anterolateral horizontal plate) and the MOL approach (PM and PL plates)

**Fig. 11:**
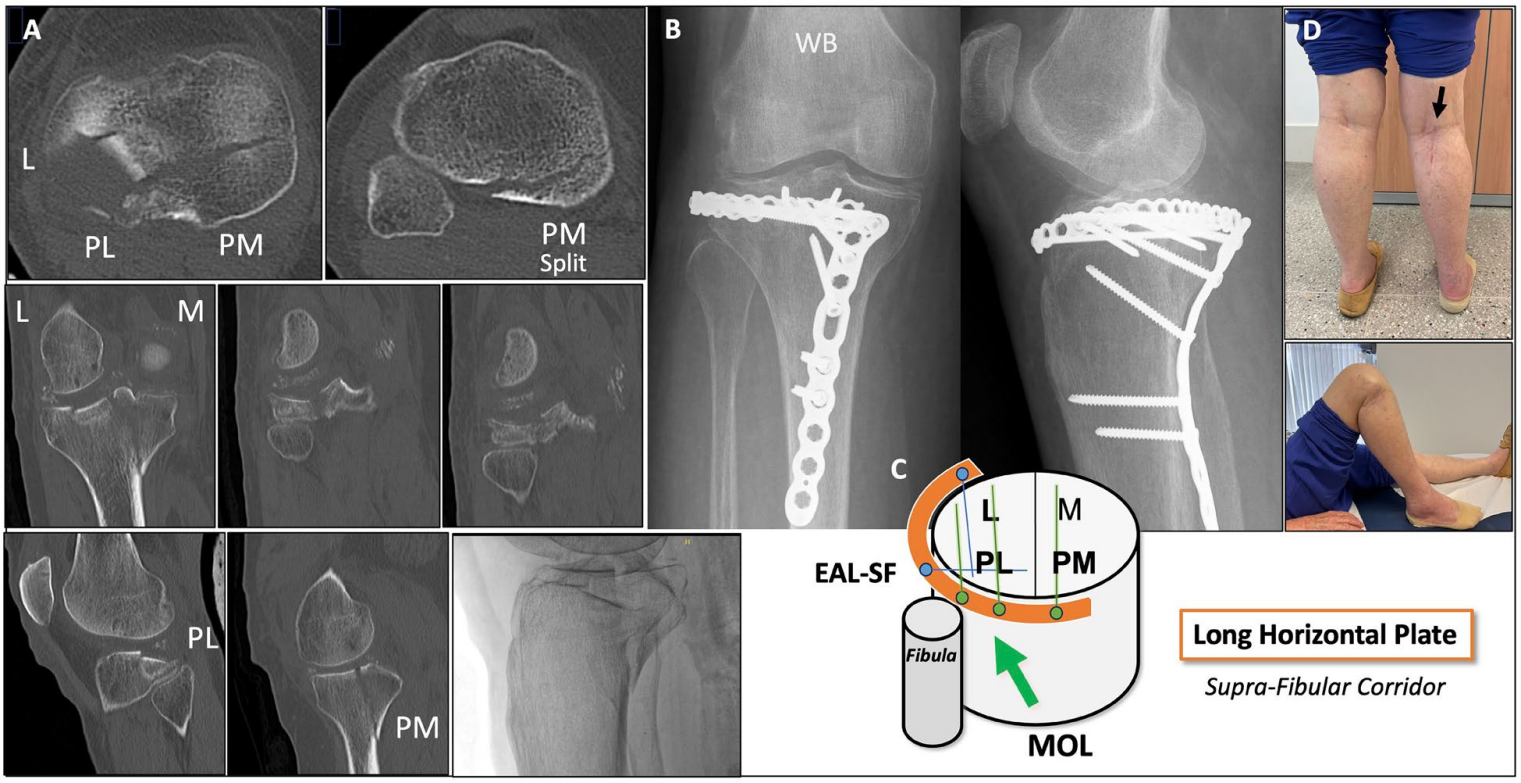
Case example of a long horizontal supra-fibular belt plate, combining the MOL approach with the supra-fibular (SF) window of the extended anterolateral (EAL) approach in a fixed prone semi-lateral position. **A** Pre-operative CT scan: three-column fracture with multiple depressed fragments in the PL and lateral columns. Calcified menisci can be seen on CT. **B** the 3 month post-operative weight-bearing radiograph. Horizontal supra-fibular plate, from posteromedial to lateral, using a straight 2.7 mm contoured locking plate. Direct reduction of the PL column and PL column fixation with posterior screws through the horizontal plate, were performed via the MOL approach. PM column split fracture was fixed with an additional PM plate. **C** Diagram representing the horizontal plate (orange) and the possibilities for screw fixation from the MOL approach (green circles) and the EAL approach (blue circles). **D** Clinical images of the patient 3 months after surgery. Black arrow: MOL incision

- Position: two main positions can be used for this approach.
  o Prone position when a single MOL approach is used (Fig. 1).
  o Prone semi-lateral position (with a bump under the ipsilateral iliac crest and table tilted) when a simultaneous anterolateral or extended anterolateral (EAL) approach is needed [4] (Fig. 6).
- Landmarks: the posterior midline (based on the Achilles tendon and fluoroscopy), the knee joint line (marked using fluoroscopy) and the fibular head are drawn (Fig. 1A).
- Skin incision: the longitudinal oblique skin incision starts 1 cm proximal to the joint line, just medial to the posterior midline and progresses distally towards medial, running parallel to the ‘theorical path’ of the MSCN (Fig. 1A, D).
- Fascia incision: before opening the medial gastrocnemius fascia, in the posterior midline, the fat tissue (between the gastrocnemius muscles) and the small saphenous vein (SSV) can be recognized by translucency (Fig. 1B). The fascia incision starts just proximal to the joint level and just medial to the SSV and progresses distally in an oblique manner towards medial, in line with the skin incision. It is important to leave some millimeters of fascia parallel to the ‘theorical path’ of the MSCN to avoid direct closure over the vein or nerve (Fig. 1C, D). Reference sutures are placed at both sides of the fascia to facilitate final closure.
- Deep posteromedial interval: medial blunt dissection in the space between the fascia and the medial gastrocnemius muscle is performed carefully, ensuring that MSCN and SSV remain lateral to the deep approach. Deep posteromedial interval between the medial gastrocnemius muscle and the pes anserinus is developed (Fig. 2A). The medial gastrocnemius muscle is retracted laterally with a blunt retractor to expose the fascia over popliteus and semimembranosus muscles (Fig. 2B). In this moment, flexion of the knee can relax the medial gastrocnemius muscle. It is not necessary to identify the popliteal neurovascular bundle, located in the interval between gastrocnemius and popliteus muscles, during the surgical approach (Fig. 3).
- Popliteus elevation: a longitudinal incision in the popliteus–semimembranosus fascia is performed in the medial border of the popliteus muscle (Fig. 4A, B). Normally, the inferior medial genicular artery (IMGA) is located under this fascia at the superior border of the popliteus. Identification and ligation of this transversal vessel is recommended (Fig. 4C, F). A second fascia incision between the popliteus and semimembranosus is performed proximal and parallel to the border of the popliteus. Adding both fascia incisions, a T-shape is drawn (Fig. 4A, D). Subperiosteal elevation of the popliteus, and part of the tibial origin of the soleus and tibialis posterior muscle is performed, looking for the lateral edge of the metaphyseal tibia (Fig. 4E). A first blunt lever retractor (Hohmann) is placed under the popliteus and the tibialis posterior muscle at the lateral edge of the tibia in the distal part of the approach. Careful placement and management of this retractor is essential to avoid damaging the ATA (Figs. 3, 4E, F).
- Posterolateral column exposure: subperiosteal dissection is performed to access the proximal lateral aspect of the tibial plateau over the fibular head, where a second lever retractor is placed (supra-fibular Hohmann; Fig. 5). The preferred retractor in this location is a bent pointed Hohmann (Fig. 6). The authors recommend avoiding continuous tension of this retractor.
- Final exposure and PL column management: the whole posterior aspect of the proximal tibia can be potentially exposed, including the PM column, the posterior cruciate ligament and the PL column (Fig. 5). The posterior joint capsule can be opened in both the PM and PL areas if necessary, but taking care to respect the posterior cruciate ligament and semimembranosus insertions. For the surgical management of the PL column through this approach, it can be beneficial to reduce the PL column before fixing the PM column, taking advantage of the PM fracture window. Many fractures can be considered similar to a ‘posterior split-depression’ (‘PM column split + PL column depression’), and therefore we suggest a similar strategy to any split-depression fracture, reducing first the PL joint fragments (Figs. 6 and 7). In particular cases of two-column PM + PL or in multi-columnar fractures, when the Main Deformity Direction (MDD) of the global fracture is towards posteromedial (PM-MDD), the PL column may present specific deformities that could be managed appropriately through this MOL approach [4]. The posterolateral lateral (PLL) segment can be intact or angulated towards the medial segment. Using this approach, this segment can be visualized and elevated in a proper direction. In these fractures, depressed articular fragments on the posterolateral central (PLC) segment can typically be found in front of the field of vision, and can be managed easily from posterior [4] (Figs. 7 and 8). PL column provisional fixation with K-wires can be performed from posterior and/or from lateral (Fig. 8). PL column definitive fixation can be obtained with posterior plates through the MOL approach (Figs. 6, 9 and 10) and/or lateral screws or plates placed in the most posterior aspect of the lateral tibial plateau through the percutaneous **(**Fig. 8B**)** or EAL approach (Fig. 6) [4]. In selected cases involving multiple depressed PL fragments, the MOL approach can be combined with the supra-fibular window of the EAL approach [4]. This enables the use of a long horizontal belt plate (from PM to lateral), allowing fixation with proximal PL screws through the MOL approach (Fig. 11).
- Closure: the popliteus muscle is reattached to the medial border of the tibia or to the fibers of medial collateral ligament, if necessary. Closure of the medial gastrocnemius fascia is performed from distal to proximal, without entrapping the SSV or the MSCN. Sometimes the proximal part of the fascia is left opened owing to the fragility of the fibres at this level and to prevent scarring or compression over the SSV and MSCN. Finally, subcutaneous and skin closure is performed.

## Results

The cadaveric study allowed the investigation of the relationship of the MOL approach with the anatomical structures at risk, which are not usually identified in the clinical practice.

The SSV and the MSCN were observed under the fascia; both structures remained lateral to the oblique fascia incision. At the level of the knee joint line, the MSCN was in the posterior midline, under the SSV, in the five specimens. Distally, the SSV remained in the posterior midline, while the MSCN was observed progressing towards medial in an oblique manner, separating from the SSV, in four specimens. The point where the MSCN starts medializing was at 5–15 mm below the level of the fibular head. In only one case, the MSCN remained in the posterior midline under the SSV, resulting in a greater ‘safe distance’ from the fascia incision. In all specimens, a correct minimum ‘safe distance’ of 10 mm, at the level of fibular head and below, was consistently observed between the fascia incision and the MSCN, being greater the distance in the distal part of the approach (Fig. 1).

The popliteal neurovascular bundle and the ATA were present in the interval between gastrocnemius and popliteus muscles (Fig. 3), being protected with the popliteus elevation during the surgical approach (Fig. 4). Evaluating the ATA and surrounding structures in the crossing area, this artery was not in direct contact with the lateral border of the tibia, being closer to the fibula than to the tibia. The popliteus muscle (ventrally) and the tibial origin of the tibialis posterior muscle (ventrally and medially) offered protection to the ATA during placement of the distal lever retractor (Fig. 4).

The exposure area, after the placement of the proximal lever retractor in the lateral tibia over the fibular head, included the whole posterior cortex, obtaining adequate proximal exposure of the PM and PL columns, including the PLC and PLL segments. The achieved exposure was considered the ‘complete exposure area’, consistently extending from corner to corner of the proximal posterior cortex, according to the defined criteria of this study, in all five cadaveric specimens (Fig. 5**)**.

## Discussion

The decision-making approach in complex tibial plateau fractures can be particularly controversial when PL and PM columns are involved [1, 4, 11, 12, 14, 16, 18, 19, 31]. In our opinion, the management of the PL column depends especially on the associated column(s) and the Main Deformity Direction (MDD), understood as a theoretical vector of global columns displacement or deformity [4]. Especially in two-column PM + PL and multi-columnar PM-MDD fractures, we believe that an optimized deep posteromedial interval approach could potentially offer a better direction to manage the deformity of the PL and PM columns in these cases [4]. In this sense, the MOL approach was designed to improve the access to the PL column through a deep posteromedial interval [4].

Several approaches, with different skin and fascia incisions and positions, have been previously described through the deep posteromedial interval between medial gastrocnemius and pes anserinus [1, 4, 8, 9, 34]. The classical posteromedial approach in the supine position uses a longitudinal skin incision 2 cm posterior to the posteromedial border of the tibia [7, 35]. This approach can be useful for simple metaphyseal PM column fractures. However, the knee flexion and the location of the incision can be a limitation when treating more complex PM column fractures [4, 9]. The Lobenhoffer approach uses a longitudinal straight incision slightly posterior, along the border of the medial gastrocnemius, performed in prone position with knee extension [8, 9]. This approach improves the management of PM column [9]. However, in our opinion, the medial location of the skin and fascia incision can limit access to the PL column. Krause et al. [11] in a cadaveric study, using the Lobenhoffer approach, refer to visibility of only fractions of the posterolateral central (PLC) segment, but a valid labelling of these regions was not consistently possible. The Luo approach (posterior reverse L-shaped approach) in prone position adds to the longitudinal incision of the Lobenhoffer approach, with a transverse incision from the centre of the popliteal fossa and develops a full thickness fasciocutaneous flap [1, 10]. Several authors agree that the Luo approach can improve access to the PM and PL column [1, 23]. Nevertheless, other authors referred limitations in the PL column exposure using the Luo approach [12, 13, 31, 32]. Orapiriyakul et al. [12], in a cadaveric study, considered a blinded area for the Luo approach when the fracture of the PL column is located more than 43.72% lateral to the lateral tibia spine. Additionally, the correct screw direction or even the capacity of placing the proximal screws of the posterior plate, when fixing the PL column, may be considered difficult using Lobenhoffer or Luo approaches [3, 12, 16]. Owing to these limitations, several authors recommend to associate specifically designed posterolateral approaches [20, 22, 25] to address the PL column even in two-column PM + PL fractures [11, 12].

In this cadaveric study using the MOL approach, we were able to obtain a successful and safe exposure of the PL and PM columns. The incision near to the posterior midline avoids the limitation for PL column access generated by the skin and fascia with the traditional Lobenhoffer approach [3] and avoids the extensive flap of the Luo approach [12, 16]. The oblique incision also optimizes access to the PL column at the proximal level, even potentially allowing perpendicular screw placement (Figs. 9 and 10), while maintaining a good exposure to the PM column.

Specifically designed posterolateral approaches through extended anterolateral (EAL), lateral or posterolateral incisions [3, 4, 20, 22–28] can be excellent options to address the PL column in certain situations, not discussed in this paper, such as one-column PL or two-column L + PL fractures [4]. However, in two-column PM + PL fractures or in multi-columnar PM-MDD fractures, the MOL approach can constitute a favourable option to manage the PM and PL columns. It can offer better control of the deformities (Fig. 7) without requiring additional posterolateral approaches [4]. These posterolateral approaches also have their own limitations and risks, including potential injury to the lateral collateral ligament, the common peroneal nerve, the lateral sural cutaneous nerve and especially the anterior tibial artery which represents the distal limit for exposure and plating in these approaches [13, 22, 29]. In this regard, the MOL approach offers the additional advantage to overcome the distal limitation imposed by the anterior tibial artery in posterolateral approaches. This allows for a complete exposure of the PL column, from the articular level to distal fragments, without limitations on the use of longer posterolateral plates (Figs. 6 and 10). In certain complex multi-columnar fractures, especially in PL-MDD fractures, both the MOL approach and the EAL approach can also serve as complementary options for PL column management [4].

Other authors have proposed direct posterior approaches between gastrocnemius muscles to address PL and PM columns [31–33]. These approaches require a more complex dissection and manipulation of the MSCN and popliteal neurovascular bundle. Motor branches of the tibial nerve can also be potentially damaged when separating the gastrocnemius muscles [36, 37]. Chouhan et al. [33], in their series of 22 patients, performing a midline gastrocnemius splitting approach (with a lazy-S shaped skin incision), reported two cases of sural nerve paresthesia. Berber et al. [31] described an approach with an inverted L-shaped skin incision similar to the Luo approach but with a transverse incision from lateral to medial, along the knee crease. The neurovascular structures were identified in the midline between gastrocnemius muscles, followed by retraction or tenotomy of the medial gastrocnemius muscle. In their series of 16 patients, they reported one patient with tibial nerve neuroapraxia, one case of partial sensory neuroapraxia and two cases of arthrofibrosis requiring manipulation [31]. Tenotomy of the medial gastrocnemius muscle is considered an option to improve PL access in posteromedial approaches [31, 38, 39]. However, several authors refer that this tenotomy should be avoided, if possible, owing to unnecessary risk of weakening the gastrocnemius-soleus muscle, late equinus deformity or knee stiffness [3, 12]. In the present study, no cadaveric specimens required medial gastrocnemius tenotomy.

Direct dissection of the popliteal neurovascular bundle is not required in the MOL approach (or in other deep posteromedial interval approaches). Normally, the elevation of the popliteus muscle protects the popliteal artery and the ATA, but anatomical variants should be considered [13]. Several studies reported that 0.8–6% of cases with a pattern where the ATA runs through the anterior surface of the popliteus muscle [40, 41]. In these infrequent situations, the artery could be at risk during the popliteus elevation if the dissection is not performed carefully. In the MOL approach, during the placement of the first Hohmann retractor, the ATA could also potentially be at risk at the level of the crossing, located at 35.7 ± 9.0 mm (range 17–50 mm) distal to the fibular head [29]. Therefore, the safest level for this retractor would be below 50 mm. However, even at the level of the foramen, the ATA can be partially protected during the placement of the retractor by the tibial origin of the tibialis posterior muscle and the location of the artery closer to the fibula (Fig. 4). The ATA runs between the two heads of the tibialis posterior muscle and continues through the interosseous foramen to enter the anterior compartment [42]. At the foramen, the ATA is located at a mean distance of 4.2 ± 0.5 mm from the lateral border of the tibia and 1.5 ± 0.4 mm from the medial border of the fibula [42]. Nevertheless, the mobility of the ATA is relatively poor at this level [32], so careful management of this retractor is essential to avoid vascular damaging.

The risk of damage to cutaneous nerves are part of iatrogenic complications in surgical approaches [43] and are often underdiagnosed [44]. In the Luo and traditional Lobenhoffer approaches, owing to a more medial incision, the saphenous nerve can potentially be damaged [3, 10, 44, 45]. In contrast, Van den Berg et al. [2] commented on the possibility of increasing the risk of damaging the sural structures by performing the incision more lateral. Berwin et al. [34] proposed a straight skin and fascia incision over the middle of medial gastrocnemius, without neurological complications in a series of six patients. In the proposed MOL approach, one of the keys is the anatomical rationale for the location and obliquity of the incision based on the anatomy of the medial sural cutaneous nerve (MSCN) and the cutaneous innervation of the posterior part of the knee [36, 37, 44–48]. The MSCN is the most medial structure of the sural nerve complex [46]. While the lateral sural cutaneous nerve has a variable origin and course, and even bifurcating branches [45, 47], the MSCN is normally located at the posterior midline under the SSV at the level of the knee joint [36, 46]. Distally, at or under the level of the fibular head, the MSCN can be found in the posterior midline, slightly lateral or usually slightly medial [45, 46]. In relation to this, we assume this possible medial path as the ‘theorical path’, which represents the most lateral limit for our skin and fascia incision (Figs. 1 and 2). The oblique fascia incision allows a proper safety margin to protect the MSCN, also preventing scarring over the nerve. Lateral retraction of the MSCN, without direct dissection of the nerve, is theoretically safe, as this nerve ends distally in a posterolateral direction, normally forming the sural nerve [47].

Regarding patient positioning, in two-column PM + PL fractures we recommend the prone position to perform the MOL approach. However, in PM or PL MDD multi-columnar fractures involving the lateral column we suggest the prone semi-lateral position to achieve direct control of the deformity and simultaneous global fracture control [4]. A relevant advantage of the MOL approach, owing to the location of the skin incision, is the possibility to combine it with anterolateral or EAL approaches in this fixed position [4], without requiring a ‘floating position’ [1] or changing from prone to supine position [17, 39]. In this fixed position, the Lobenhoffer approach or the medial longitudinal incision of the Luo approach would be very difficult to perform. This proposed simultaneous control strategy could be advantageous not only for global fracture management, but also for individual PL column management when the MOL approach is combined with the EAL approach [4]. Firstly, some PL column fractures could benefit from simultaneous reduction from the MOL approach while performing provisional or definitive fixation from the EAL approach (Fig. 6). Secondly, in complex cases, especially in multi-columnar PL-MDD fractures, reduction of the PL column can be managed and/or controlled simultaneously through both approaches. Subsequently, fixation of the PL column, including comminuted distal PL cortex fragments, can be performed using long vertical plates through the MOL approach (Fig. 10). Furthermore, in cases with multiple depressed articular fragments, this strategy also enables the use of long horizontal belt plates (from posteromedial to lateral) through the ‘supra-fibular corridor’ from the MOL approach (Fig. 11). This technique expands the applications of the horizontal plating concept [18, 19, 23, 24, 49, 50], offering a novel alternative that allows direct control and proximal screw fixation of the PL column through the MOL approach.

This study has some potential limitations. The anatomical study was based only on five cadaveric specimens. Nevertheless, our anatomical findings were consistent with those reported in literature. The exposure area was not directly compared with other approaches in this cadaveric study. Nonetheless, complete exposure of the PL and PM column was obtained, in contrast to other comparable cadaveric studies that reported only partial PL column exposure using other deep posteromedial interval approaches [11, 12]. The cadavers may have less soft tissue tension than living patients, so the exposure area could differ from clinical practice. However, the feasibility of this approach in living patients is reinforced by several complementary clinical case examples outlined in the figures of this paper. Therefore, we emphasize the importance of an appropriate learning curve, taking into account all the technical details described in this paper. Additional large-scale specific clinical studies are required to corroborate the safety and effectiveness of this approach.

The strength of this study is that it presents a detailed description of this approach with anatomical rationale for this modification. Additionally, potential clinical benefits for treating complex fractures are described in detail and reinforced by case examples. We also suggest novel options for PL column management through this approach, especially in PM-MDD multi-columnar fractures.

## Conclusions

On the basis of the present cadaveric study, the MOL approach can be considered a feasible option to access PL and PM columns through a deep posteromedial interval. The location of the skin and fascia incision, the deep approach and the retractor placement can be considered safe with respect to cutaneous nerves and deep neurovascular structures. Successful exposure of the whole PM and PL columns, including the PLC and the PLL segments, can be obtained. Additionally, the possibility of performing this approach in a fixed prone semi-lateral position can also facilitate simultaneous anterolateral or EAL approaches in multi-columnar fractures.

## Data Availability

The data and materials are available from the corresponding author upon reasonable request.

## Abbreviations

ATA: Anterior tibial artery
EAL: Extended anterolateral
IMGA: Inferior medial genicular artery
MDD: Main deformity direction
MOL: Modified oblique Lobenhoffer
MSCN: Medial sural cutaneous nerve
PL: Posterolateral
PLC: Posterolateral central
PLL: Posterolateral lateral
PM: Posteromedial
SSV: Small saphenous vein
